# Pyroptosis in urinary malignancies: a literature review

**DOI:** 10.1007/s12672-023-00620-7

**Published:** 2023-01-26

**Authors:** Sheng Wang, Xinyang Liao, Xingyu Xiong, Dechao Feng, Weizhen Zhu, Bojue Zheng, Yifan Li, Lu Yang, Qiang Wei

**Affiliations:** 1 The Department of Urology, West China Hospital, Sichuan University, No. 37 Guoxue Xiang, Chengdu, 610041, Sichuan China; 2 The Department of Clinical Medicine, West China Medical School, Sichuan University, Chengdu, China

**Keywords:** Pyroptosis, Urinary malignancies, Gasdermins, Targeted therapy

## Abstract

Urinary neoplasms refer to malignant tumours occurring in any part of the urinary system, including the kidney, renal pelvis, ureter, bladder, prostate, etc. The worldwide incidence of urinary system tumours has been increasing yearly. Available methods include surgical treatment, radiotherapy, chemotherapy, endocrine therapy, molecular targeted therapy, and immune therapy. In recent years, emerging evidence has demonstrated that cell pyroptosis plays an important role in the occurrence and progression of malignant urinary tumours. Pyroptosis is a new type of cell death that involves inflammatory processes regulated by gasdermins (GSDMs) and is characterized by membrane perforation, cell swelling and cell rupture. Recent studies have shown that pyroptosis can inhibit and promote the development of tumours. This manuscript reviews the role of pyroptosis in the development and progression of prostate cancer, kidney cancer and bladder cancer and introduces the latest research results in these fields to discuss the therapeutic potential of the pyroptosis pathway in urinary malignancies.

## Background

In 2020, the overall number of new cases of malignant tumours of the urinary system estimated in the United States of America reached 159,120 and 33,820 deaths [1]. The most common urinary malignancies are kidney, bladder, and prostate cancers. Overall, further research on the occurrence and development mechanism of urological malignant tumours is needed. Many studies [2–5] have noted that the mode of cell death plays an important role in the occurrence and development of tumours. As one of the modes of cell death, the role and mechanism of pyroptosis in urinary system tumours deserve further study.

Cell death correlates closely with infectious diseases, nervous system diseases, autoimmune diseases, cardiovascular diseases, tumours, and other human diseases [6–9]. The modes of cell death can be divided into unregulated accidental cell death (ACD) and regulatory cell death (RCD), which can be regulated through elaborate molecular mechanisms. Under specific physiological conditions, RCD is also called programmed cell death (PCD) [10]. Apoptosis, necroptosis, and pyroptosis are all categorized as RCD [10]. The process of apoptosis is characterized by the caspase cascade, with caspase-3 being the main response enzyme. Necroptosis is a caspase-independent RCD, and RIPK3/MLKL is its key pathway [11]. Pyroptosis was first named by Cookson and Brennan in 2001 [12], who found that the death of macrophages infected with Salmonella typhimurium dependent on Caspase-1 [13]. Expansion of knowledge on pyroptosis has prompted research, suggesting that pyroptosis is a potential target in the treatment of urinary malignancies.

## Molecular mechanism of pyroptosis

### The canonical pathway of pyroptosis

When the body becomes infected or immunized, cells activate and release all kinds of inflammasomes, put forward by Martinon et al. in 2002, which is a key part of innate immunity and an important molecular complex for activating caspase 1, which mediates the classical pathway of pyroptosis [14]. There are three types of inflammasomes according to the structural characteristics of the sensors: the nucleotide-binding oligomerization domain-like receptor family (NLRP1, NLRP3, NLRC4, NLRC5), AIM2-like receptor (ALR) and pyrin proteins. After recognizing a series of pathogen-associated molecular patterns (PAMPs) or danger-associated molecular patterns (DAMPs) [15], inflammasome sensors bind to adapters on apoptosis-associated speck-like proteins (ASCs) to activate caspase-1, and then gasdermin D (GSDMD) is cleaved as a cutting substrate, resulting in pyroptosis [16]. One exception is NLRC4, in which the step of ASC binding after recognition with DAMPs or PAMPs is skipped, with direct activation of caspase-1 to promote pyroptosis [17].

### The noncanonical pathway of pyroptosis

Lipopolysaccharide, the cell wall component of gram-negative bacteria that invade humans or mice, is a typical inflammatory stimulator that binds with mouse caspase-11 and human caspase-4 and caspase-5, followed by direct cleavage of GSDMD into an N-terminal fragment (31 kDa) and a C-terminal fragment (22 kDa). The N-terminal fragment forms extensive air-dissolved pores on the cell membrane by dissolving phospholipids or cardiolipins, which promote release of IL-1β and IL-18 and eventually cause pyroptosis. On the other hand, the C-terminal fragment inhibits pyroptosis by binding with the N-terminal fragment [18].

In addition, activated Caspase-4, 5, and 11 activates the channel protein Pannexin-1 on the cell membrane, which is mainly involved in regulating and controlling the entry and exit of small molecular substances in and out of cells. Once activated, Pannexin-1 promotes release of potassium ions and ATP, and the potassium ions activate the NLRP3 inflammasome and promote release of IL-1β. Moreover, ATP activates and opens the P2 × 7 channel on the cell membrane, further destroying its integrity and completing pyroptosis [15].

### Other caspase-mediated pyroptosis processes

**Fig. 1:**
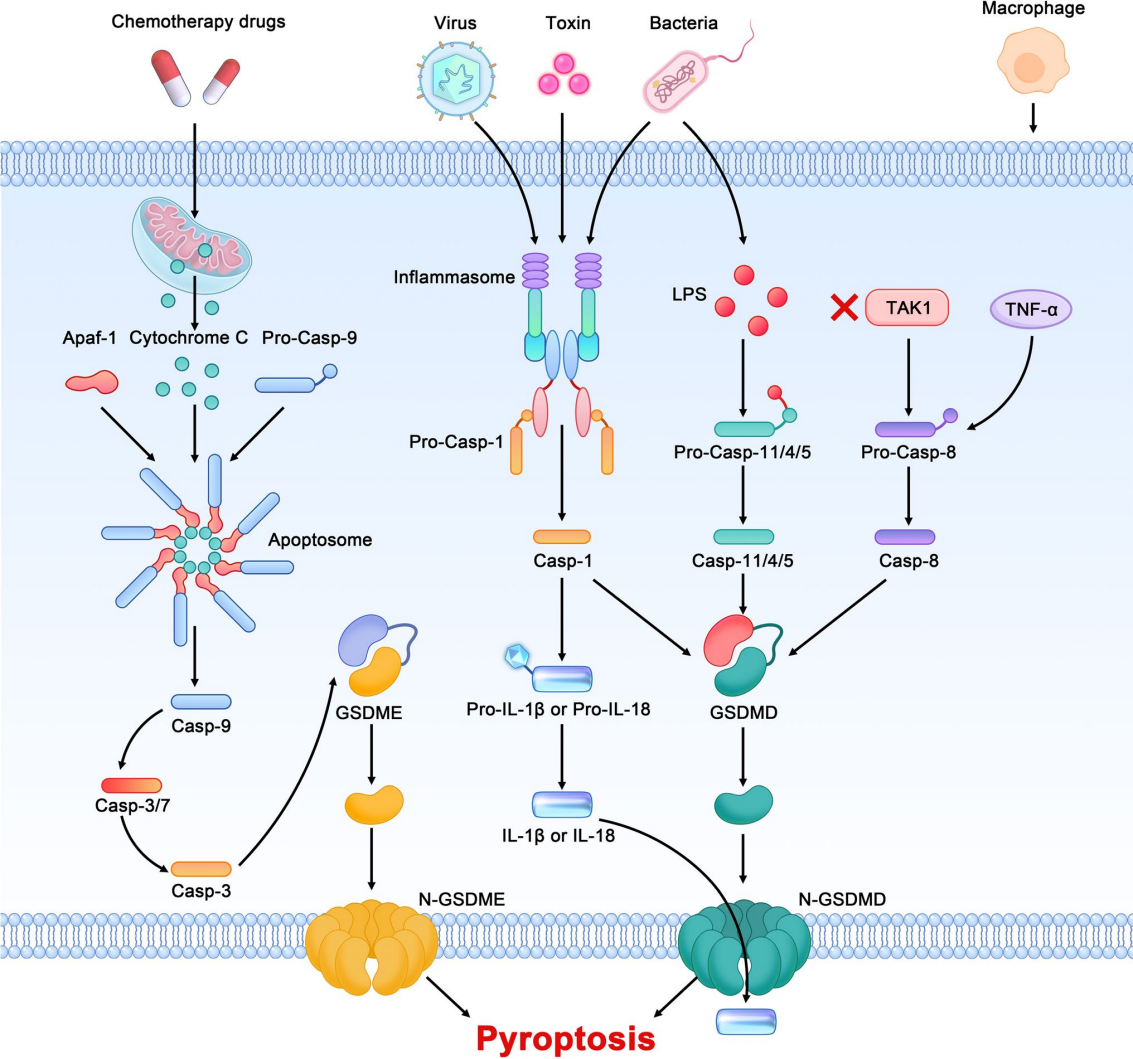
Molecular mechanisms of the canonical inflammasome, noncanonical inflammasome and other caspase-mediated processes in pyroptosis

In addition to the above-mentioned pyroptosis processes mediated by caspase-1, 4, 5 and 11, some studies have suggested that chemotherapeutic drugs activate caspase-3 and cleave Gasdermin E (GSDME), with the N-terminal fragment perforating the membrane and destroying its osmotic barrier, resulting in pyroptosis [19–21]. Tumour necrosis factor and Toll-like receptors 3 and 4 can inhibit IAPs (inhibitor apoptosis proteins) and TAK1 (transforming growth factor β activated kinase 1), leading to activation of caspase-8 and cleavage of GSDMD in macrophage [22]. The specific mechanisms of pyroptosis are shown in Fig. 1.

## Pyroptosis and urinary malignancies

### Pyroptosis in prostate cancer

Prostate cancer (PCa) is one of the most common malignant tumours in men. For locally advanced or metastatic prostate cancer, traditional endocrine therapy can only benefit patients in the short term. After a period of continuous endocrine treatment, hormone-sensitive prostate cancers (HSPCs) usually progress to castration-resistant prostate cancers (CRPCs), with poor quality of life and poor prognosis. To achieve targeted and accurate therapy for prostate cancer, its pathogenesis warrants further investigation.

The occurrence and development of prostate cancer is accompanied by changes in related gene pathways, and many signalling pathways are involved in the proliferation, migration, and apoptosis of prostate cancer cells. Some studies suggested that pyroptosis is involved in abnormal changes in signalling pathways in prostate cancer; the main pyroptosis pathways related to prostate cancer are the Caspase-1 pathway, Caspase-4/5/11 pathway and Caspase-3 signalling pathway [19, 23].

As a classical inflammatory signalling pathway, the Caspase-1 pathway plays a crucial role in promoting pyroptosis in prostate cancer cells. Through in vitro cell lines and in vivo animal subcutaneous tumorigenesis experiments, Xu Z et al. found that NLRP3 expression was up-regulated in prostate cancer tissues and cell lines [24]. NLRP3 overexpression promoted malignant progression of PCa cell lines, while NLRP3 knockdown inhibited it through positive regulation of caspase 1. Their results revealed that the NLRP3 inflammasome plays a crucial role in promoting the malignant progression of PCa by activating caspase 1 [24].

Ponomareva L et al. reported that AIM2 mRNA expression levels are significantly decreased in prostate tumours compared with the normal prostate, with even basal expression levels of AIM2 protein being undetectable in some prostate cancer cell lines [25]. Moreover, the cytoplasmic DNA (derived from pathogens, necrotic cells, or senescent cell-derived exosomes) existing in normal primary human prostate epithelial cells (PrECs) and prostate cancer (PC-3 cell line) can activate the AIM2 inflammasome [25]. This suggests that the expression patterns of Caspase-1 and the AIM2 inflammasome might have prognostic significance for disease progression in prostate cancer.

Karan et al. reported that increased expression of NLRP12 in prostate cancer shows that it might play a crucial role in activating the NF-κB and IL-1β signalling pathways and that it might be associated with the occurrence and progression of prostate cancer [26]. They indicated that the NLRP12 inflammasome related to pyroptosis can upregulate Caspase-1 and downstream IL-1 β and IL-18 to promote the occurrence and progression of prostate cancer.

Many studies have shown that lipopolysaccharide (LPS) is involved in the proliferation, invasion, and metastasis of prostate cancer cells [27–29]. Recently, it has been found that intracellular LPS can activate the Caspase-4/5/11 pathway, induce pyroptosis, and inhibit tumour growth. In prostate cancer cells, changes in the structure of the endoplasmic reticulum lead to an increase in expression of Caspase-4, which in turn induces programmed cell death [30]. Additionally, in vitro experiments have suggested that cardiotonic glycosides might diminish the stability of prostate cancer cells, induce an increase in Caspase-4 expression, and promote pyroptosis. Nishizaki et al. found that the anticancer drug HUHS1015 can activate Caspase-4 and its downstream pathway as well as inhibit cell proliferation in prostate cancer cell lines, including DU145, LNCap and PC-3 [31]. Malafa et al. found that vitamin E has an anti-tumour effect by inducing expression of Caspase-4 [32]. A retrospective study showed that mRNA expression of Caspase-5 was significantly elevated in patients with prostate cancer, indicating that Caspase-5-involved pyroptosis may be related to the risk of PCa [33].

In contrast to other pyroptosis pathways, the one mediated by Caspase-3 is associated with GSDME protein cleavage and is often activated by traditional chemotherapy or targeted drug therapy. Studies have shown that expression of Caspase-3 in prostate cancer tissue is significantly decreased [34]. Long non-coding RNA (lncRNA) plasmacytoma variant translocation 1 (PVT1) is involved in the occurrence and progress of prostate cancer. Knockout of PVT1 can significantly up-regulate expression of Caspase-3 in mouse prostate cancer tissue [35]. In summary, there is a close relationship between the Caspase-3 pathway and prostate cancer.

### Pyroptosis in renal cell carcinoma

Renal carcinoma is a serious public health problem, with an estimated 338,000 new cases and 144,000 deaths worldwide each year [36]. There is already evidence that the only curative treatment is surgery for localized renal cell carcinoma (RCC), which is the most common type of kidney cancer. Unfortunately, approximately one-third of patients treated with surgery have distant relapses, and once the disease progresses, overall prognosis is poor [37, 38].

Increasing evidence has confirmed that pyroptosis plays a crucial role in cancer [4, 39, 40]. Whether pyroptosis promotes or inhibits tumours is controversial, and its potential role in clear cell renal cell carcinoma (ccRCC) treatment efficacy and prognosis is not fully understood. Previous studies have suggested that cell death plays a dual role in renal cell carcinoma [41]. Liver X receptor (LXR) is a member of the nuclear receptor superfamily of DNA-binding transcription factors [42]. Bobin-dubigeon et al. [43] demonstrated that LXR-α promotes tumour metastasis in RCC by inhibiting NLRP3 inflammasome-dependent pyroptosis. As a natural compound, ursolic acid upregulates expression of NLRP3 in RCC [44] and then activates caspase-1, which eventually causes prolapse of renal cell carcinoma and inhibits tumour growth [45]. In addition, recent studies have shown that inhibition of BRD4 in RCC suppresses cell proliferation as well as epithelial–mesenchymal transition (EMT) progression and plays an anti-tumour role in renal cell carcinoma by activating the NF-κB-NLRP3-Caspase-1 signalling pathway [46].

Interestingly, it has also been found that pyroptosis can promote the occurrence and development of renal cell carcinoma. Recent studies have showed that expression of most pyroptosis regulatory genes correlates positively with the prognosis of ccRCC, and four kinds of pyroptosis regulatory factors have been found during ccRCC tumorigenesis, indicating that their enhanced expression is associated with the poor prognosis of renal cell carcinoma [47, 48]. Involvement of AIM2 in various tumours has been proven; nonetheless, Zhang et al. reported a clear cancer-promoting effect in ccRCC [47]. Furthermore, Peng et al. found that arsenic can induce renal cell carcinoma by promoting the AIM2 inflammasome and increasing release of IL-1β and IL-18 [49]. Tang et al. also revealed that FOXD2-AS1 affects the key genes Gasdermin B (GSDMB) and NLRP1 [50]. This suggests that FOXD2-AS1 may be a new therapeutic target in renal cell carcinoma therapy, and the clinicopathological manifestations of pyroptosis-related lncRNAs were also revealed in this investigation.

### Pyroptosis in bladder malignancies

The fourth most common and eighth most fatal malignant tumour among men in the United States is bladder cancer [1]. The results from He et al. showed that GSDMB can bind with STAT3 to increase its phosphorylation level, promoting transcription of GSDMB and increasing expression of genes related to glucose metabolism (*HK2, LDNA, ENO2, IGFBP3*), with enhanced proliferation of bladder cancer cells [51]. In addition, they confirmed that the interaction between USP24 and GSDMB can prevent degradation of GSDMB in bladder cancer cells [51]. The USP24/GSDMB/STAT3 axis may become a new signalling pathway for the treatment of bladder tumours.

Chen et al. showed that pyroptosis has a certain impact on non-immunoreactive tumours, and there is a significant correlation between the immune activity of tumours and pyroptosis in bladder cancer [52]. Based on Kaplan‒Meier (KM) curve analysis, they showed that GSDMB and caspase-6 are associated with better prognosis in bladder cancer. Therefore, they studied the spatial distribution of these two genes in different immune types of bladder tumours. The results showed tumours with high expression of GSDMB and caspase-6 to be mainly immune inflammatory tumours, with those having poor expression of GSDMB and caspase-6 being mostly immune-desert tumours. Pancancer gene detection showed that GSDMB has a strong relationship with expression of immune checkpoints and the degree of invasion of immune cells, which further confirmed the role of GSDMB and caspase-6 in the immune system.

Zhang et al. confirmed that the serine protease granzyme B secreted by cytotoxic T cells can cleave GSDME and lead to the death of target cells [53]. Moreover, once activated, GSDME has the potential to turn a “cold” tumour into a “hot” tumour, which can be recognized and controlled by the immune system [53].

Immune checkpoint inhibitors (ICIs) are known to be more effective when combined with therapies that increase the number of CD8 + T lymphocytes [54]. Chen et al. found a significant correlation between pyroptosis and expression of immune checkpoints [52]. Previous studies have shown that patients with high expression of PD-L1 and PD-1 are usually relatively sensitive to immunosuppressive therapy [55]. Therefore, they speculate that the combination of ICIs and pyroptosis inducers might have great potential to promote the development and application of new combined therapeutic strategies and new immunotherapeutic agents [52]. In addition, some scholars have noted that pyroptosis levels can be a predictive factor for the prognosis and survival of bladder neoplasm patients [56, 57].

Collectively, pyroptosis plays an important role in the occurrence and development of urinary system tumours, and the specific promoting or inhibitory effect is shown in Table 1.

**Table 1.**
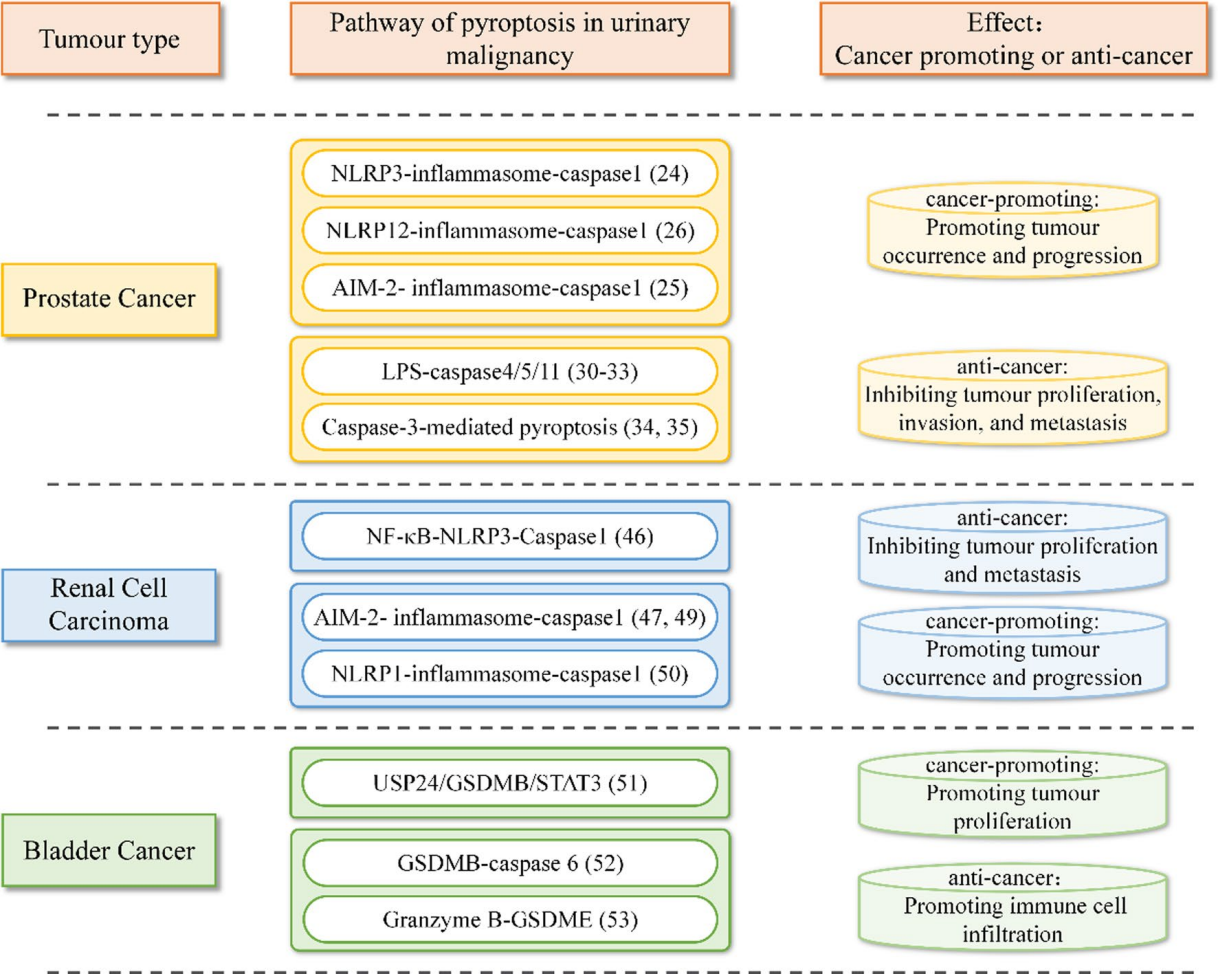
The pathway and effect of pyroptosis in urinary malignancy

## Summary and prospects

Pyroptosis is a pro-inflammatory form of programmed cell death that has become a hot research topic in recent years. Although the molecular mechanism of its occurrence has been comprehensively clarified, it is still unclear whether pyroptosis inhibits or promotes tumours of different types.

Pyroptosis can not only enhance the body’s immune response through the acute inflammation it induces, thereby inhibiting tumour cell proliferation, but also inhibit the anti-tumour immunity caused by the chronic tumour necrosis generated by a small amount of tumour cell pyroptosis in the central hypoxic area of ​​the tumour, forming a tumour cell microenvironment suitable for tumour cell growth and accelerating tumour development[58]. Therefore, the complicated mechanisms of factors related to pyroptosis inhibition and promotion of tumour initiation and progression, how to precisely regulate cancer cell pyroptosis to achieve clinical benefits, and how to reduce damage to normal cells during anti-tumour treatment still require further exploration. It is worth mentioning that pyroptosis might provide new treatment ideas and treatment targets for urinary malignancies.

## Data Availability

Not applicable.

## Abbreviations

GSDMs: Gasdermins
ACD: Accidental cell death
RCD: Regulatory cell death
PCD: Programmed cell death
PAMPs: Pathogen-associated molecular patterns
DAMPs: Danger-associated molecular patterns
ASCs: Apoptosis-associated speck-like proteins
IAPs: Inhibitor apoptosis proteins
TAK1: Transforming growth factor β activated kinase 1
RCC: Renal cell carcinoma
EMT: Epithelial–mesenchymal transition
ICIs: Immune checkpoint inhibitors
